# An Integrative Approach of Neeli Bhringraj Polyherbal Hair Oil in Clinical Evaluation of Androgenetic Alopecia (Khalitya) and Premature Hair Graying (Pālitya): A Randomized, Double-Blind Clinical Study

**DOI:** 10.7759/cureus.111988

**Published:** 2026-07-03

**Authors:** Maheshvari N Patel, Nayan Patel, Apeksha Merja, Ravikumar R Patel, Manisha Mishra

**Affiliations:** 1 Clinical Research, NovoBliss Research Private Limited, Ahmedabad, IND; 2 Pharmacology, Swaminarayan University, Gandhinagar, IND; 3 Clinical Research Operations, NovoBliss Research Private Limited, Ahmedabad, IND; 4 Dermatology, NovoBliss Research Private Limited, Ahmedabad, IND; 5 Pharmaceutics, Swaminarayan University, Gandhinagar, IND; 6 Clinical Trials, NovoBliss Research Private Limited, Ahmedabad, IND; 7 Clinical Trials, Svahita Ayurvedic Clinic, Mumbai, IND

**Keywords:** ayurvedic formulation, hair density, hair thickness, integrative medicine, neeli bhringaraj

## Abstract

Background

Androgenetic alopecia, hair fall (*Khalitya*), and premature hair graying (*Pālitya*) are common conditions with multifactorial etiology that significantly affect psychosocial well-being. In Ayurveda, these disorders are associated with Vata-Pitta doshic imbalance leading to impaired nourishment of hair follicles (*Romakupa*) and altered hair growth dynamics. *Neeli Bhringadi Taila*, a classical Ayurvedic polyherbal formulation used in *Shiro Abhyanga*, is traditionally indicated for promoting hair growth and maintaining hair pigmentation; however, systematic clinical evaluation is required to substantiate its efficacy in integrative hair care.

Methods

This randomized, double-blind, prospective clinical study was conducted over 120 days in 78 participants aged 18-55 years presenting with androgenetic alopecia, hair fall, and hair graying. Participants were randomized into three groups: polyherbal hair oil (*Neeli Bhringadi* hair oil), polyherbal hair oil with 5% minoxidil, and 5% minoxidil alone. Out of 78 enrolled participants, 75 participants (25 per arm) successfully completed the study. Efficacy assessments included hair growth rate, hair density, hair thickness, hair fall count, scalp condition, scalp hydration, anagen-to-telogen (A:T) ratio, gray hair evaluation, Adherent Scalp Flaking Score (ASFS), hair strength, and scanning electron microscopy. Safety was evaluated through clinical monitoring and adverse event reporting.

Results

All test product groups demonstrated statistically significant improvements in hair growth parameters compared with the baseline. By Day 120, participants in the polyherbal hair oil (*Neeli Bhringadi hair oil*) group demonstrated statistically significant improvements, with hair growth rate increasing to 290.29 ± 39.57 µm/day, hair density increasing to 277.60 ± 33.69 hairs/cm², and hair thickness increasing to 16.56 ± 1.98 µm. Additionally, Graying Severity Score (GSS) decreased to 9.24 ± 3.28, and ASFS decreased to 8.08 ± 4.95. All changes were statistically significant (p < 0.0001).

Conclusion

*Neeli Bhringadi* hair oil exhibited favorable safety and demonstrated potential benefits in supporting normalization of the hair growth cycle and improvement in hair pigmentation, as evidenced by a reduction in premature hair graying. The observed effects suggest a positive influence on overall hair and scalp health, likely through modulation of follicular function and scalp conditioning. These findings highlight the therapeutic potential of this Ayurvedic polyherbal formulation as an evidence-based herbal approach for managing hair fall and graying. Furthermore, its integration within routine hair care practices may offer sustained benefits, supporting its role in integrative and preventive hair care strategies.

## Introduction

Premature canities, hair graying, and androgenic alopecia are the most common psychosocial concerns affecting both women and men across different age groups. Compromised hair quality, including reduced hair density, telogen effluvium, and excessive hair shedding, may impact quality of life and self-esteem [1,2]. 

Factors such as nutritional deficiencies, oxidative stress, hormonal imbalance, psychological stress, environmental exposure, and inappropriate hair care routine contribute to progressive hair fall and altered hair growth cycles [3,4]. The multifactorial nature of hair loss necessitates therapeutic approaches that address both scalp health and hair follicle function.

Among the currently available regular therapies, topical minoxidil remains one of the most widely prescribed treatments for androgenic alopecia and hair fall. While minoxidil has illustrated efficacy in increasing the anagen phase and increasing hair density in individuals, its clinical use is often limited by changes in the response rate by individuals, its requirement for prolonged use and regular application, and recurrence of hair thinning upon discontinuation [5,6]. Moreover, adverse effects, such as dryness, scalp irritation, erythema, pruritus, dermatitis, and the initial shedding phase of hair, contribute to non-adherence and reduced compliance [7,8]. These drawbacks highlight the need for well-tolerated, safer, and holistic alternatives that support sustained hair and scalp health.

In Ayurveda, hair-related disorders are described under contexts, such as *Khalitya* and *Kesha Patana*, addressing their onset primarily to the vitiation of *Vata *and *Pitta doshas*, along with imbalanced nutrition of hair follicles (*Romakupa*) [9]. Classical Ayurvedic wordings emphasize external application (Abhyanga) of medicated oils to the scalp helps to restore *doshic *balance, enhance microcirculation, and strengthen hair roots. Neeli Bhringraj oil is a classical Ayurvedic formulation traditionally indicated for promoting hair growth, preventing hair fall, and maintaining scalp health. It commonly contains herbs, such as *Eclipta alba (Bhringraja)*, *Indigofera tinctoria *(*Neeli*),* Emblica officinalis* (*Amalaki*), and *Glycyrrhiza* *glabra* (*Yashtimadhu*), processed in a suitable oil base. These botanicals are reported to possess anti-inflammatory, antioxidant, and follicle-nourishing properties, and improve the hair growth cycle and hair graying, which may collectively support improved hair health [10-12]. The synergistic action of multiple herbs is a key principle of Ayurvedic polyherbal formulations, aiming to enhance therapeutic efficacy while minimizing adverse effects.

Recently, there has been an increased consumer and clinical interest in herbal and polyherbal hair oils as multifunctional therapies. However, many marketed ayurvedic hair oils lack clinical evidence to support their claims, and comparative results against marketed products, such as minoxidil, remain limited. A *Neeli Bhringraj* polyherbal hair oil formulation may offer a multi-targeted approach by working on hair growth, follicular nourishment, hair graying, and scalp health. In contrast, monotherapy approaches, including single-herb oils or synthetic agents, may act through limited mechanisms and fail to address the complex etiology of hair fall comprehensively [13,14]. Despite the long-term use of polyherbal *Neeli Bhringraj* hair oil, there is a scarcity of well-designed comparative clinical studies evaluating its safety and efficacy. Direct comparisons between Ayurvedic formulations and pharmacological therapy are essential to generate evidence-based insights into their clinical performance. Therefore, the present clinical study was designed to comparatively evaluate the safety and efficacy of a polyherbal hair oil, topical minoxidil, and a combination of polyherbal hair oil and minoxidil in individuals having hair fall problems, androgenic alopecia, and hair graying, with the aim of addressing this critical evidence gap and supporting integrative clinical decision-making.

## Materials and methods

Ethical conduct of the study

This clinical study was conducted in accordance with ethical standards, including the principles outlined in the Declaration of Helsinki, the International Council for Harmonisation-Good Clinical Practice (ICH-GCP) guidelines, Good Clinical Practice Guidelines for Clinical Trials in Ayurveda, Yoga and Naturopathy, Unani, Siddha and Homoeopathy (AYUSH-GCP), and the Indian Council of Medical Research (ICMR) ethical guidelines for biomedical research involving human participants. 

The clinical study protocol was approved by the ACEAS-Independent Ethics Committee on 08 June 2024 with the approval no. NB230043-NB. The study was prospectively registered with the Clinical Trials Registry of India (CTRI) with registration number CTRI/2024/06/069335 and Clinical Trial. Gov (no. NCT06552039) to ensure transparency and public accessibility of clinical study-related information. 

The study has been reported in accordance with the Consolidated Standards of Reporting Trials (CONSORT) guidelines to ensure methodological rigor, transparency, robustness, and completeness in the reporting of study design, methodology, statistical analysis, and results. 

All participants were enrolled only after providing written informed consent. The informed consent process included detailed explanations of study objectives, endpoints, visits, procedures, potential risks and benefits, confidentiality safeguards, and the voluntary nature of participation. Participant rights, safety, and well-being were protected throughout the study duration, and compensation was provided for each scheduled visit, in accordance with ethical and regulatory requirements. 

Study design

This was a prospective, randomized, double-blind, interventional, clinical study designed to evaluate the safety and efficacy of a polyherbal hair oil, a combination of polyherbal hair oil and minoxidil, and a 5% minoxidil hair serum alone.

The study was conducted at a Central Drug Standard Control Organization (CDSCO)-registered Contract Research Organization at NovoBliss Research Private Limited, Ahmedabad, India. The total product usage duration was 120 days, with continuous use of the test product as per the allocated arm.

Based on sample size determination, 78 participants were enrolled in the study, with 26 participants allocated to each arm. Of these, 75 participants (25 per arm) successfully completed the study. Within each arm, 13 participants had androgenetic alopecia (AGA), while the other 13 participants had complaints of hair fall, both having premature canitis.

The primary objective of the study was to evaluate the efficacy of the investigational products on objective hair and scalp parameters, including hair density, hair thickness, hair growth rate, scalp condition, hair strength, hair fall, scalp skin hydration, A:T ratio, Adherent Scalp Flaking Score (ASFS), and graying severity score (GSS). Secondary objectives included assessment of the general appearance of hair and scalp, scanning electron microscopy, as well as the evaluation of participant-reported outcomes using a structured product perception questionnaire (Appendices) to capture user experience, cosmetic acceptability, and overall satisfaction with the study products.

The study was conducted over a period of 120 ± 2 days and included six scheduled visits. The first participant’s first visit occurred on December 26, 2024, and the last participant’s last visit was completed on June 28, 2025. Safety and efficacy assessments were performed at baseline, Day 01, and during follow-up visits at Day 45 ± 2 days, Day 87 ± 2 days, Day 90 (three days after visit 4), and Day 120 ± 2 days.

Eligibility criteria

Eligible participants were adults aged between 18 and 55 years, including both males and non-pregnant, non-lactating females. Participants in generally good health, as determined by medical history, physical examination, and the investigator's clinical assessment, were eligible for inclusion in the study. Participants with androgenetic alopecia, premature graying, and hair thinning were included, with screening hair fall counts of approximately 40-50 hairs in females and 25-30 hairs in males. Participants were required to have stable or no hormonal or contraceptive therapy for at least six weeks before enrolment and agreed to maintain the same throughout the study.

Participants were excluded if they had hair fall due to diseased conditions, such as anemia or thyroid disorders, or if they had any other active dermatological conditions of the scalp other than hair loss or dandruff. Individuals who had undergone hair transplantation, laser therapy, or other hair growth procedures were considered likely to influence hair growth assessments during the study and were excluded. This criterion was applied to minimize the potential confounding effects on efficacy evaluations. Individuals with a history of hair transplantation, laser therapy, or other hair growth procedures were excluded, as were those who had used topical hair loss products within four weeks or systemic products within three months before screening.

Details about test product(s)

Test Product A: Polyherbal Hair Oil

The polyherbal hair oil was applied directly to the scalp using the fingertips and gently massaged in circular motions to ensure uniform distribution and facilitate follicular penetration. The oil was left on the scalp overnight and washed off the following morning using shampoo. The application was performed thrice weekly throughout the study duration.

Test Product B: Polyherbal Hair Oil in Combination With 5% Minoxidil Serum

Participants assigned to the combination arm applied polyherbal hair oil to the scalp thrice weekly, following the same application and massage procedure as described for Test Product A. In addition, a 5% minoxidil hair serum was applied once daily at night to the targeted scalp areas using a dropper. The serum was left on the scalp without rinsing.

Test Product C: 5% Minoxidil Serum

Participants in this arm applied 5% minoxidil hair serum once daily to the affected scalp regions. The serum was applied using a dropper, starting from the center of the application area and gently spreading to ensure adequate coverage. The formulation was left on the scalp without rinsing.

Neeli Bhringraj Hair Oil Formulation

The polyherbal hair oil formulation is enriched with multiple botanicals traditionally recognized in the Ayurvedic literature for their role in supporting hair and scalp health. The formulation was manufactured under controlled laboratory conditions using standardized extraction and blending procedures. The test batch was identified as batch no. NBO-101, manufactured in October 2024, with an expiry date of September 30, 2027. The product was supplied for research purposes and was used strictly in accordance with the study protocol. Product storage was conducted under controlled conditions - a cool and dry place to ensure consistency throughout the study duration (Table 1).

**Table 1:** Formulation Ingredient List Composition of the polyherbal formulation, listing all ingredients used in the Neeli Bhringadi hair oil. Rasa: fresh herbal juice extract, Ksira: milk or milk-derived liquid used during processing, and Taila: Oil base (sesame oil used as a carrier). The final volume of the prepared formulation was approximately 600-650 mL.

No.	Ayurvedic name (API)	Botanical/common name	Part used	Quantity
1	Nili Rasa	Indigofera tinctoria	Leaf juice	768 mL
2	Bhringraj Rasa	Eclipta alba	Plant juice	768 mL
3	Satakratu Lata Rasa	Cardiospermum halicacabum	Plant juice	768 mL
4	Dhatri Phala Rasa	Emblica officinalis	Fruit juice	768 mL
5	Aja Ksira	Goat milk	Milk	768 mL
6	Narikela Ksira	Cocos nucifera	Coconut milk	768 mL
7	Mahisi Ksira	Buffalo milk	Milk	768 mL
8	Dhenudugdha	Cow milk	Milk	768 mL
9	Tila Taila (Murcchita)	Sesamum indicum	Seed oil	768 mL
10	Yaṣṭimadhu	Glycyrrhiza glabra	Root	32 g
11	Gunja	Abrus precatorius	Root	32 g
12	Anjana (Rasanjana)	*Berberis* spp.	Extract	32 g

The formulation was prepared using pharmacopeial-grade raw materials in accordance with the procedures outlined in the Ayurvedic Formulary of India. Initially, sesame oil was subjected to the *Murcchana *process, a traditional Ayurvedic oil-processing technique involving purification and conditioning of the base oil to enhance its stability, reduce undesirable odor, and improve its suitability for medicinal formulation preparation, thereby obtaining *Murcchita Tila Taila*. Fresh plant materials of *Nili Bhṛngaraja*, *Kakatika*, and *Amalaki *were thoroughly washed, separately ground, and filtered through a muslin cloth to obtain their respective fresh juices. Fresh coconut endosperm was cleaned, ground, and expressed through a muslin cloth to obtain coconut milk. Goat milk, buffalo milk, and cow milk were individually strained to remove impurities.

Solid herbal ingredients, namely *Yaṣṭimadhu*, *Gunja*, and *Anjana*, were cleaned, shade-dried, pulverized separately, and passed through a 180 µm sieve to obtain a fine powder. These powders were triturated with an adequate volume of water to prepare a homogeneous herbal paste (*kalka*).

The murcchita sesame oil was transferred to a stainless-steel vessel and heated gently. The prepared kalka was added in increments with continuous stirring, followed by the gradual addition of respective herbal juices. The mixture was heated for approximately three hours with constant stirring, maintaining the temperature between 50°C and 90°C during the initial heating phase. Heating was then discontinued, and the mixture was allowed to stand overnight.

On the subsequent day, coconut milk, goat milk, buffalo milk, and cow milk were added sequentially, and the heating process was continued intermittently over a period of three days. The progress of oil preparation was monitored using classical *Sneha Siddhi* *Laksaṇas*, including assessment of the *kalka *consistency by rolling between the fingers. Upon completion, the oil was filtered, allowed to cool, and stored in airtight containers under controlled conditions until further use [15]. The final volume of the prepared formulation was approximately 600-650 mL.

Evaluation methods

Standardization of Hair Assessment

Hair assessments were consistently performed on a single, predefined area of the scalp to ensure accuracy and reproducibility of data. A 1 cm² region was marked using medical-grade ink to create a temporary tattoo, serving as a fixed reference point throughout the study duration [16].

C*ASLite Nova - Hair Density and Thickness, Hair Growth Rate, Scalp Condition*

Hair thickness and density were measured using the CASLite Nova instrument. For thickness assessment, images were captured at 50× magnification under the "Thickness" tab of the Phototrichogram menu, ensuring the visibility of follicles and scalp. A minimum of three to four hair strands were selected using the drag-and-mark technique to calculate the average thickness [17]. At 60× magnification, the CASLite Nova Hair Analyzer (Catseye Systems and Solutions Pvt. Ltd., Mumbai, India) was used to measure the hair growth rate using the phototrichogram technique. Images were acquired on Day 01 and Day 4, and three randomly chosen hairs were analyzed to determine the daily hair growth rate (µm/day) from the change in length over the three-day interval. Scalp condition was evaluated using CASLite Nova, which analyzes phototrichogram images to assess dryness, flaking, and texture. The reported “keratin” values reflected optical indicators of scalp surface condition, not biochemical keratin levels. A decrease in values indicated improvement.

Graying Severity Score

Canities were quantitatively assessed using a standardized phototrichogram-based method. The scalp was divided into five regions: frontal, vertex, bilateral temporal, and occipital. In each region, a 1 cm² area with maximum graying was identified, trimmed to approximately 1 mm hair length, and imaged for hair count analysis. The percentage of gray hairs was calculated for each area and scored as follows: Score 1 (<10% gray hair/cm²), Score 2 (10-30%), and Score 3 (>30%). The total GSS was obtained by summing the scores across all five regions (range: 0-15) and categorized as mild (0-5), moderate (6-10), or severe (11-15) [18,19].

Hair Pull Test

The hair pull test was performed by gently grasping approximately 20-60 hairs between the thumb, index, and middle fingers close to the scalp and applying a firm, controlled traction away from the scalp [19].

The 60-Second Hair Combing Test - Hair Fall

The sixty-second hair count method was employed to assess hair shedding over a 60-second combing period. Participants were instructed to flip their hair forward and comb from the back to the front of the scalp over a contrasting-colored sheet for 60 seconds, using a wide-toothed study comb [17].

General Appearance of Hair and Scalp

The general appearance of hair and scalp was evaluated by a dermatologist and a trained clinical evaluator using a standardized clinical grading system. Key hair attributes, including hair volume, density, plasticity, shine, smoothness, oiliness, dryness, and strength, were assessed visually and by palpation to determine the overall hair quality. Scalp characteristics, such as itchiness, dryness, redness, roughness, and scaliness, were also systematically evaluated. This combined expert assessment provided a comprehensive overview of hair health and scalp condition.

Hair Pluck Test - Percentage Anagen and Telogen Hairs

Approximately 30 hairs were plucked using protected forceps while maintaining scalp tension. The hairs were mounted on a slide, and bulbs were examined at 40× magnification to count anagen and telogen hairs.

Hair Root Strength

Hair root strength was assessed using a standardized hair pull test, wherein a bundle of approximately 20-60 hairs was gently pulled from the scalp. The number of extracted hairs was recorded and used to qualitatively grade hair root strength as poor, average, or good. A reduction in the number of hairs extracted over time indicated improved hair root strength.

Adherent Scalp Flaking Score

Adherent scalp flaking was assessed using a standardized grading method in which the scalp was divided into eight predefined zones. Each zone was evaluated for the presence of dandruff flakes adhering to the scalp surface and scored on a scale of 0-10 (0 = no flakes, 10 = excessive adherent flaking). Loose flakes present in the hair were not considered during assessment. The total ASFS was calculated by summing the scores across all eight zones, yielding a possible score range of 0-80. Based on the total score, severity was categorized as mild (16-24), moderate (25-34), and severe (35-80). A reduction in ASFS over time was interpreted as an improvement in scalp condition [20].

Scalp Skin Hydration

Scalp hydration was measured using a Corneometer® CM 825 (Courage+Khazaka Electronic GmbH; Cologne, Germany), a capacitance-based device that quantifies moisture content of the stratum corneum. Measurements were taken at a predefined scalp site under standardized conditions. The device measures changes in dielectric properties associated with skin hydration, with higher values indicating greater moisture content. An increase in hydration values over time was interpreted as an improvement in scalp hydration and barrier function. Scalp hydration was measured using a Corneometer® CM 825.

Hair Shine

Hair shine was measured using Glossymeter GL 200 (Courage+Khazaka Electronic GmbH; Cologne, Germany), which evaluates surface gloss based on light reflection. The device emits light at a 60° angle and measures both directly reflected and diffusely scattered light from the hair surface. Higher gloss values indicate greater shine and improved cuticle alignment, while increases over time reflect enhancement in hair surface smoothness and appearance.

Scanning Electron Microscopy

Scanning electron microscopy (SEM) was employed to evaluate microstructural changes in hair fibers following the use of the assigned test products. The SEM analysis focused on assessing cuticle morphology, including cuticle alignment, surface smoothness, cuticle lifting, erosion, and overall structural integrity of the hair shaft [21]. 

Safety and Product-Related Adverse Events

Product-related adverse events (AEs) were systematically monitored throughout the study period. Participants were evaluated for signs of redness, irritation, pruritus, or other localized scalp reactions through both self-reported feedback and clinical scalp examinations conducted at each study visit.

Statistical analysis

All data were reviewed before analysis to ensure accuracy and completeness. Records with missing data were excluded from the analyses. Continuous outcome measures (e.g., hair density, hair growth rate, hair thickness, A:T ratio, and tensile strength) were described using descriptive statistics, including the number of observations (N), mean, standard deviation (SD), median, minimum, and maximum values. Categorical outcomes (e.g., dermatological assessments and questionnaire responses) were summarized using frequencies and percentages, with graphical presentation where appropriate.

For within-participant comparisons of continuous variables from baseline to post-baseline, either a paired t-test (parametric) or the Wilcoxon signed-rank test (non-parametric) was applied, depending on data distribution. Between-group comparisons of continuous variables were conducted using either the independent samples t-test (parametric) or the Mann-Whitney U test (non-parametric), as appropriate. For ordinal categorical outcomes, Wilcoxon signed-rank tests were used to compare baseline and post-product assessments, and between-group comparisons were conducted using Mann-Whitney U tests when applicable. A two-sided significance level of 5% was applied for all statistical tests.

All statistical analyses were performed using IBM SPSS Statistics for Windows, version 29.0.1.0 (released 2023; IBM Corp., Armonk, NY, USA) and Microsoft Excel 2019 (Microsoft Corp., Redmond, WA, USA).

Sample Size Determination

The sample size for the study was determined based on a two-sample t-test power calculation. Assuming a two-sided significance level (α) of 0.05 and a statistical power (1−β) of 0.80, a standardized effect size (Δ) of 0.80 and a standard deviation of 1.2 were considered. Based on these assumptions, a minimum of 25 participants per product arm was required to detect a clinically meaningful difference between groups. To account for an anticipated attrition rate of approximately 10%, a total of 78 participants (26 participants per arm) were planned for enrolment, ensuring that at least 75 participants (25 participants per arm) would complete the study and provide sufficient power to meet the study objectives.

Randomization and Blinding

Participants were randomized in a 1:1:1 allocation ratio into three product groups: polyherbal hair oil, polyherbal hair oil with 5% minoxidil, and minoxidil alone. The randomization sequence was generated using the R Software (version 4.3.1, 64-bit) by an independent biostatistician, ensuring unbiased and concealed group assignment. To maintain the study’s double-blind design, all test products were packaged in identical containers, and both participants and outcome assessors remained unaware of group assignments. Study staff responsible solely for product dispensing were not involved in any other study-related procedures, minimizing the risk of unblinding. All statistical analyses and randomization processes were performed using the R Software (R Core Team, 2023; R Foundation for Statistical Computing, Vienna, Austria).

## Results

Participant demographics and baseline characteristics

**Figure 1:**
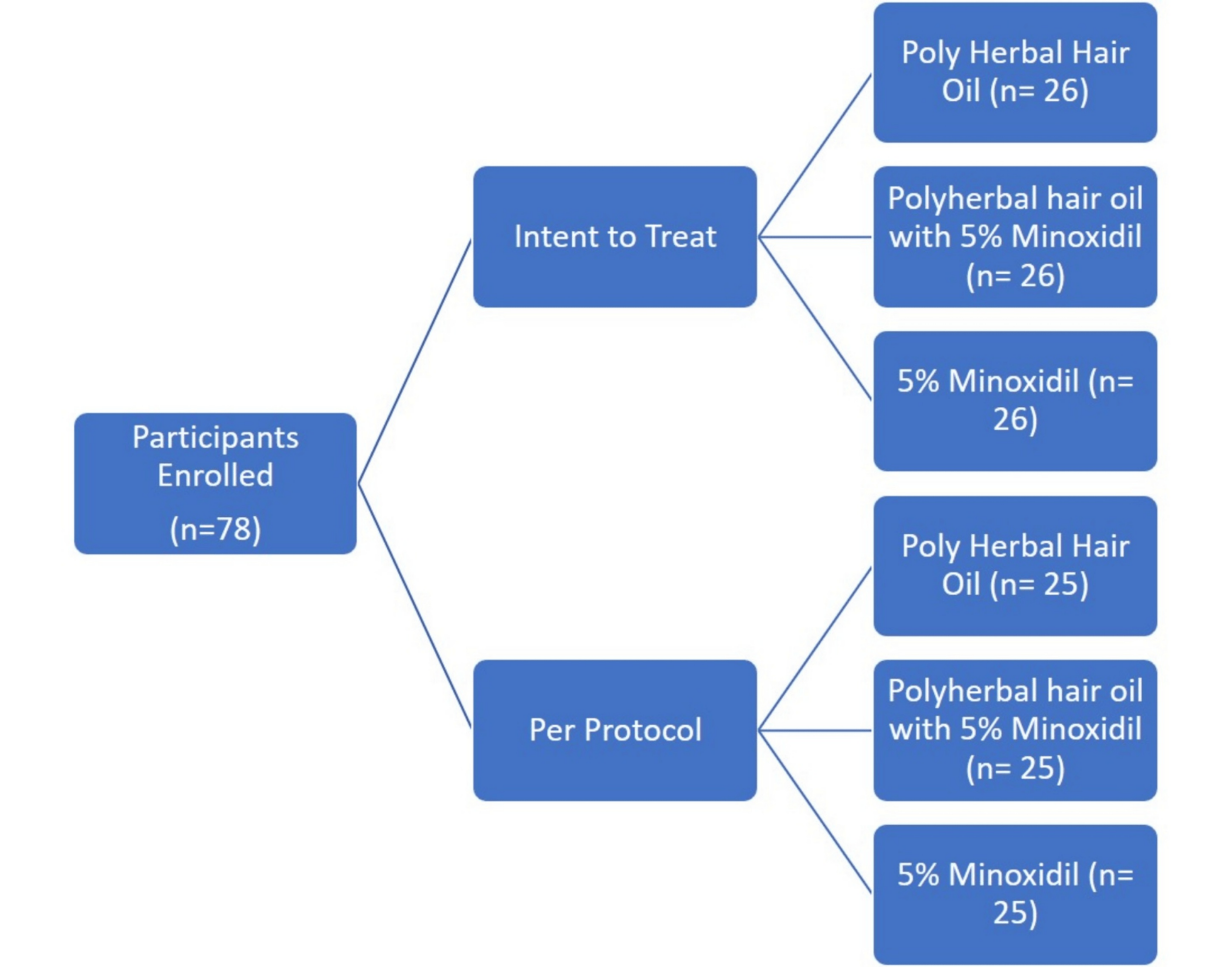
Participant Disposition Flowchart Flow diagram depicting participant enrolment, randomization, allocation, follow-up, and analysis throughout the study period.

The study comprised a total of 78 participants, including 32 females and 46 males. The mean age of the participants was 42.19 ± 7.26 years (Figure 1).

Hair growth rate

**Figure 2:**
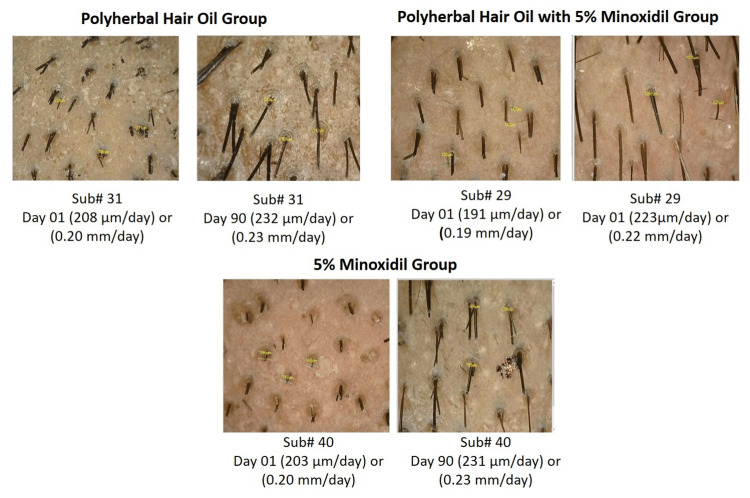
Hair Growth Rate Changes in hair growth rate across study groups over the treatment period. Sub# denotes anonymized participant identification number. Sub# 31 represents a participant from the polyherbal hair oil group, Sub# 29 represents a participant from the polyherbal hair oil with minoxidil group, and Sub# 40 represents a participant from the minoxidil group. For subject# 31, Day 01 (208 µm/day), Day 90 (232 µm/day). For subject# 29, Day 01 (191 µm/day), Day 90 (223 µm/day). For subject# 31, Day 01 (203 µm/day), Day 90 (231 µm/day). At 60× magnification, the CASLite Nova Hair Analyzer was used to measure the hair growth rate using the phototrichogram technique. Images were acquired on Day -4 and then on Day 01, and similarly on Day 7 and then on Day 90. Three randomly chosen hairs were analyzed to determine the daily hair growth rate (µm/day) from the change in length over the three-day interval.

In the polyherbal hair oil group, the mean hair growth was found to be 249.88 ± 41.33 µm (0.25 ± 0.04 mm) at baseline; it increased to 290.29 ± 39.57 µm (0.29 ± 0.04 mm) or 17.05% on Day 90, with a statistically significant p-value of <0.0001. In the polyherbal with minoxidil group, the mean hair growth was found to be 231.92 ± 50.44 µm (0.23 ± 0.05 mm) at baseline; it increased to 286.21 ± 54.58 µm (0.28 ± 0.05 mm) or 24.36% on Day 90, with a statistically significant p-value of <0.0001. In the minoxidil group, the mean hair growth was found to be 233.84 ± 49.72 µm (0.23 ± 0.05 mm) µm at baseline; it increased to 263.96 ± 48.59 µm (0.26 ± 0.05 mm) or 14.13% on Day 90, with a statistically significant p-value of <0.0001 (Figure 2).

Hair thickness

**Figure 3:**
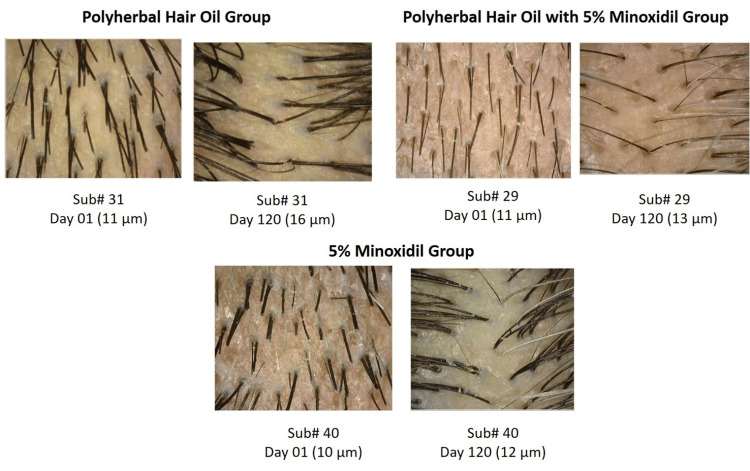
Hair Thickness Changes in hair thickness across study groups over the treatment period. Sub# denotes anonymized participant identification number. Sub# 31 represents a participant from the polyherbal hair oil group, Sub# 29 represents a participant from the polyherbal hair oil with minoxidil group, and Sub# 40 represents a participant from the minoxidil group. For subject# 31, Day 01 (11 µm), Day 90 (16 µm). For subject# 29, Day 01 (11 µm), Day 90 (13 µm). For subject# 31, Day 01 (10 µm), Day 90 (12 µm).

In the polyherbal hair oil group, the mean hair thickness was found to be 13.20 ± 2.02 at baseline, and it increased to 16.56 ± 1.98 or 26.36% on Day 120 with a statistically significant p-value of <0.0001. In the polyherbal with minoxidil group, the mean hair thickness was found to be 11.16 ± 2.72 at baseline, and it increased to 15.04 ± 2.46 or 38.99% on Day 120, which was statistically significant with a p-value of <0.0001. In the minoxidil group, the mean hair thickness was found to be 12.04 ± 1.86 at baseline, and it increased to 14.56 ± 2.12 or 21.64% on Day 120, which was statistically significant with a p-value of <0.0001 (Figure 3).

Hair density

**Figure 4:**
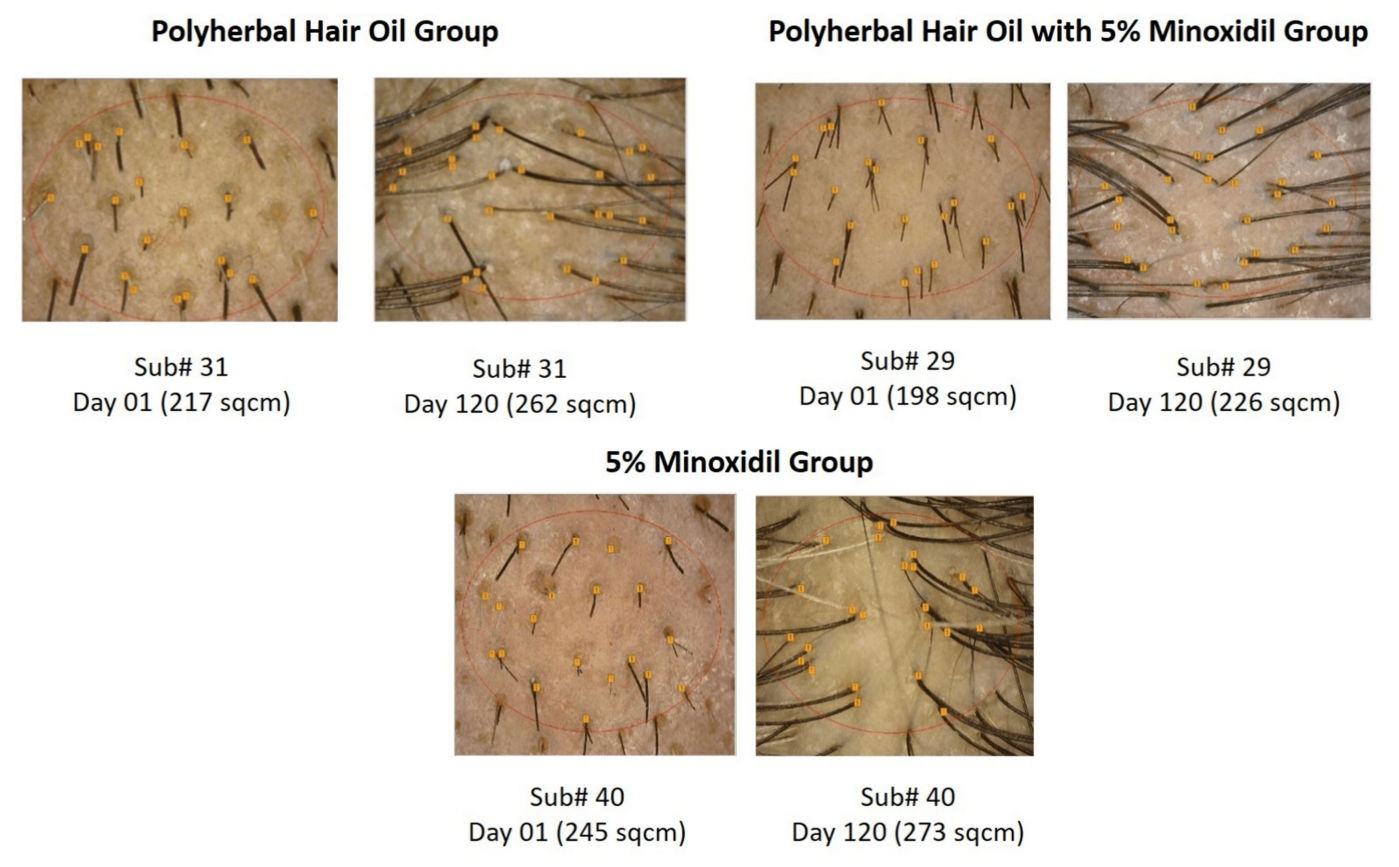
Hair Density Changes in hair density across study groups over the treatment period. Sub# denotes anonymized participant identification number. Sub# 31 represents a participant from the polyherbal hair oil group, Sub# 29 represents a participant from the polyherbal hair oil with minoxidil group, and Sub# 40 represents a participant from the minoxidil group. For subject# 31, Day 01 (217 cm^2^), Day 90 (262 cm^2^). For subject# 29, Day 01 (198 cm^2^), Day 90 (226 cm^2^). For subject# 31, Day 01 (245 cm^2^), Day 90 (273 cm^2^).

In the polyherbal hair oil group, the mean hair density was found to be 240.08 ± 33.58 at baseline, and it increased to 277.60 ± 33.69 or 16.24% on Day 120, which was statistically significant with a p-value of <0.0001. In the polyherbal with minoxidil group, the mean hair density was found to be 230.32 ± 43.67 at baseline, and it increased to 269.52 ± 37.93 or 18.46% on Day 120, which was statistically significant with a p-value of <0.0001. In the minoxidil group, the mean hair density was found to be 226.08 ± 29.42 at baseline, and it increased to 257.88 ± 29.36 or 14.52% on Day 120, which was statistically significant with a p-value of <0.0001 (Figure 4).

Scalp skin hydration

In the polyherbal hair oil group, the mean value was 9.67 ± 1.04 at baseline, and it increased to 12.08 ± 1.07 or 25.43% on Day 120 with statistically significant improvements with p-values of <0.0001. In the polyherbal with minoxidil group, the mean value was 9.71 ± 1.52 at baseline, and it increased to 12.53 ± 1.62 or 30.30% on Day 120 with a statistically significant p-value of <0.0001. In the minoxidil group, the mean value was 9.71 ± 1.11 at baseline, and it increased to 11.75 ± 1.06 or +21.78% on Day 120, with a statistically significant p-value of <0.0001.

Adherent Scalp Flaking Score

In the polyherbal hair oil group, the mean value of the ASFS was 22.64 ± 5.96 at baseline, and it decreased to 8.08 ± 4.95 or 63.67% by Day 120, with a statistically significant p-value of <0.0001. In the polyherbal with minoxidil group, the mean value was 22.64 ± 6.85 at baseline, and it decreased to 5.76 ± 5.08 or 75.88% by Day 120, with a statistically significant p-value of <0.0001. In the minoxidil group, the mean value was 21.12 ± 6.22 at baseline, and it decreased to 9.36 ± 5.77 or 54.49% by Day 120, with a statistically significant p-value of <0.0001.

Graying Severity Score 

**Figure 5:**
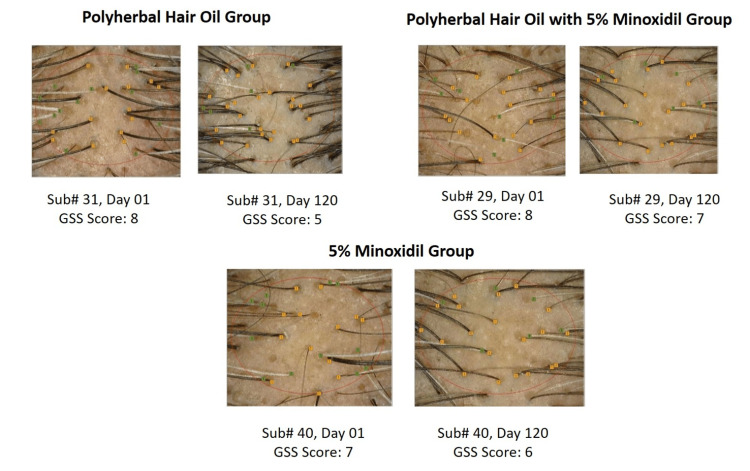
Graying Severity Score Changes in hair graying severity score across study groups over the treatment period. Sub# denotes anonymized participant identification number. Sub# 31 represents a participant from the polyherbal hair oil group, Sub# 29 represents a participant from the polyherbal hair oil with minoxidil group, and Sub# 40 represents a participant from the minoxidil group. For subject# 31, Day 01 (8), Day 90 (5). For subject# 29, Day 01 (8), Day 90 (7). For subject# 31, Day 1 (7), Day 90 (6).

In the polyherbal hair oil group, the mean value was 11.92 ± 2.90 at baseline, and it decreased to 9.24 ± 3.28 or 24.18% by Day 120, with a statistically significant p-value of <0.0001. In the polyherbal with minoxidil group, the mean value was 12.20 ± 2.22 at baseline, and it decreased to 10.40 ± 2.97 or 15.99% by Day 120, which was also statistically significant with a p-value <0.0001. In the minoxidil group, the mean value was 12.44 ± 2.75 at baseline, and it decreased to 12.00 ± 3.08, representing a 4.28% reduction by Day 120 with a statistically significant p-value of <0.01 (Figure 5).

Changes in evaluation parameters, including hair density, hair thickness, skin hydration, ASFS score, GSS, and scalp condition by CASLite Nova, were evaluated from baseline to the end of the study period to determine the overall effect of the intervention. The findings demonstrated the efficacy and performance of the test product over time (Tables 2-3).

**Table 2:** Change in Evaluation Parameters from Baseline to the End of Study Duration Values are expressed as mean ± SD. n = 25 participants per group. %CFB = percentage change from baseline. Units: hair density (hairs/cm²), hair thickness (µm), scalp hydration (arbitrary units), ASFS (0-80 cumulative score), and GSS (score). Reported p-values correspond to within-group comparisons versus baseline. The statistical test (paired t-test) was used. Participants who completed the final visit were included in the analysis, while data from intermediate visits were analyzed based on availability. SD: standard deviation; ASFS: adherent scalp flaking score; GSS: Graying Severity Score

Variables	Visit day	Poly herbal oil					Poly herbal oil + minoxidil					Minoxidil				
Statistics		N	Mean ± SD	%CFB	p-value	T-value	N	Mean ± SD	%CFB	p-value	T-value	N	Mean ± SD	%CFB	p-value	T-value
Hair density (hairs/cm²)	Day 01	25	240.08 ± 33.58	-			25	230.32 ± 43.67	-			25	226.08 ± 29.42	-		
	Day 45	25	250.56 ± 32.35	4.54%	<0.0001	12.35	22	246.64 ± 40.66	4.72%	<0.0001	14.42	24	234.83 ± 29.01	4.51%	<0.0001	24.31
	Day 90	24	262.38 ± 29.32	9.71%	<0.0001	13.92	24	255.25 ± 38.49	12.83%	<0.0001	10.22	25	248.36 ± 29.01	10.21%	<0.0001	7.44
	Day 120	25	277.60 ± 33.69	16.24%	<0.0001	9.46	25	269.52 ± 37.93	18.46%	<0.0001	13.41	25	257.88 ± 29.36	14.52%	<0.0001	9.59
Hair thickness (µm)	Day 01	25	13.20 ± 2.02	-			25	11.16 ± 2.72	-			25	12.04 ± 1.86	-		
	Day 45	25	14.32± 1.95	8.82%	<0.0001	-4.77	22	12.45 ± 2.40	11.43%	<0.0001	9.72	24	12.67 ± 1.90	5.07%	<0.001	4.90
	Day 90	24	15.42 ± 2.02	18.05%	<0.0001	-4.40	24	13.54 ± 2.59	23.60%	<0.0001	11.87	25	13.52 ± 1.94	12.66%	<0.0001	10.36
	Day 120	25	16.56 ± 1.98	26.36%	<0.0001	-4.46	25	15.04 ± 2.46	38.99%	<0.0001	16.15	25	14.56 ± 2.12	21.64%	<0.0001	10.55
Skin hydration (a.u.)	Day 01	25	9.67 ± 1.04	-			25	9.71 ± 1.52	-			25	9.71 ± 1.11	-		
	Day 45	25	10.69 ± 1.15	10.67%	<0.0001	9.35	22	10.81 ± 1.44	12.76%	<0.0001	8.24	24	10.56 ± 0.97	9.96%	<0.0001	7.82
	Day 90	24	11.40 ± 1.22	18.73,	<0.0001	-4.29	24	11.54 ± 1.38	20.04%	<0.0001	8.86	25	11.16 ± 1.08	15.48%	<0.0001	11.8
	Day 120	25	12.08 ± 1.07	25.43%	<0.0001	-4.37	25	12.53 ± 1.62	30.30%	<0.0001	12.65	25	11.75 ± 1.06	21.78%	<0.0001	13.96
ASFS scoring	Day 01	25	22.64 ± 5.96	-			25	22.64 ± 6.85	-			25	21.12 ± 6.22	-		
	Day 45	25	16.64 ± 3.45	23.39%	<0.0001	-5.92	22	16.73 ± 3.78	20.97%	<0.0001	-5.28	24	17.58 ± 3.87	11.89%	0.0003	-4.274
	Day 90	24	13.75 ± 4.62	36.67%	<0.0001	-7.06	24	14.33 ± 6.48	37.80%	<0.0001	-4.12	25	15.92 ± 3.98	21.24%	<0.001	-3.933
	Day 120	25	8.08 ± 4.95	63.67%	<0.0001	-11.26	25	5.76 ± 5.08	75.88%	<0.0001	-4.39	25	9.36 ± 5.77	54.49%	<0.001	-4.208
GSS	Day 01	25	11.92 ± 2.90	-			25	12.20 ± 2.22	-			25	12.44 ± 2.75	-		
	Day 45	25	11.08 ± 2.94	6.99%	0.0012	-3.31	22	11.82 ± 2.42	1.39%	0.1858	-0.53	24	12.25 ± 2.69	0.56%	0.1573	-1.41
	Day 90	24	10.17 ± 2.91	13.59%	<0.0001	-3.63	24	11.17 ± 2.70	8.70%	<0.0001	-1.85	25	11.84 ± 2.91	4.97%	0.0079	-2.66
	Day 120	25	9.24 ± 3.28	24.18%	<0.0001	-4.23	25	10.40 ± 2.97	15.99%	<0.0001	-3.91	25	12.00 ± 3.08	4.28%	0.0084	-2.64

**Table 3:** Scalp Condition by CASLite NOVA Scalp condition based on keratin. Participants who completed the final visit were included in the analysis, while data from intermediate visits were analyzed based on availability.

Product	Score	Visit 2 (Day 01)	Visit 3 (Day 45)	Visit 5 (Day 90)	Visit 6 (Day 120)
		N= 25	N=25	N=24	N=25
Poly herbal oil	Dry scalp with some keratin	15 (60%)	23 (92%)	19 (79.17%)	11 (44%)
	Dry scalp with much keratin	10 (40%)	2 (8%)	0 (0%)	0 (0%)
	Normal scalp in good condition - hair density and thickness	0 (0%)	0 (0%)	5 (20.83%)	14 (56%)
		N = 25	N = 22	N = 24	N = 25
Poly herbal oil + minoxidil serum	Dry scalp with some keratin	20 (80%)	21 (95.45%)	20 (83.33%)	4 (16%)
	Dry scalp with much keratin	5 (20%)	0 (0%)	0 (0%)	0 (0%)
	Normal scalp in good condition - hair density and thickness	0 (0%)	1 (4.55%)	4 (16.67%)	21 (84%)
		N = 25	N = 24	N = 25	N = 25
Minoxidil serum	Dry scalp with some keratin	15 (60%)	21 (87.5%)	22 (88%)	12 (48%)
	Dry scalp with much keratin	10 (40%)	3 (12.5%)	0 (0%)	0 (0%)
	Normal scalp in good condition - hair density and thickness	0 (0%)	0 (0%)	3 (12%)	13 (52%)

Hair root strength

In the polyherbal hair oil group, on Day 01, 72% of participants were graded as having poor hair root strength, while 28% were rated average, and none were rated good. By Day 45, a shift was observed with only 28% rated as poor and 72% as average, with no participants achieving a good rating. On Day 90, 79.17% of participants were rated average and 20.83% good, with no poor scores remaining. By Day 120, a marked improvement was noted with 84% of participants rated as good and only 16% as average.

In the polyherbal with minoxidil group, on Day 01, 64% of participants were rated poor and 36% as average, with none achieving a good rating. On Day 45, the poor rating dropped to 40.91%, while 50% were average, and 9.09% achieved a good rating. By Day 90, only 4.17% were still rated poor, 83.33% were average, and 12.5% had improved to good. By Day 120, a significant improvement was observed, with 96% of participants rated as good and only 4% as average.

In the minoxidil group, on Day 01, 48% of participants were rated poor, 44% as average, and 8% as good. By Day 45, the proportion of poor scores reduced to 37.5%, the average increased to 58.33%, and a good rating was observed in 4.17%. On Day 90, all poor ratings were eliminated, with 76% rated average and 24% good. By Day 120, 80% of the participants achieved good hair root strength, while 20% remained in the average category.

Anagen and telogen hairs

In the polyherbal hair oil group, on Day 01 at baseline, the percentage of hairs in the anagen phase was found to be 38.68 ± 11.19 at baseline. It significantly increased to 63.56 ± 11.21 on Day 120, showing a 75.42 change, with a statistically significant p-value of <0.0001. In the polyherbal with minoxidil group, the mean value was found to be 44.10 ± 13.74 at baseline, and it increased to 63.19 ± 12.93, 61.01 change from baseline on Day 120, with a statistically significant p-value <0.001. In the minoxidil group, the mean value was found to be 37.91 ± 14.39 at baseline, and it increased to 64.55 ± 11.32, 96.30 on Day 120, with a statistically significant p-value of <0.0001.

**Figure 6:**
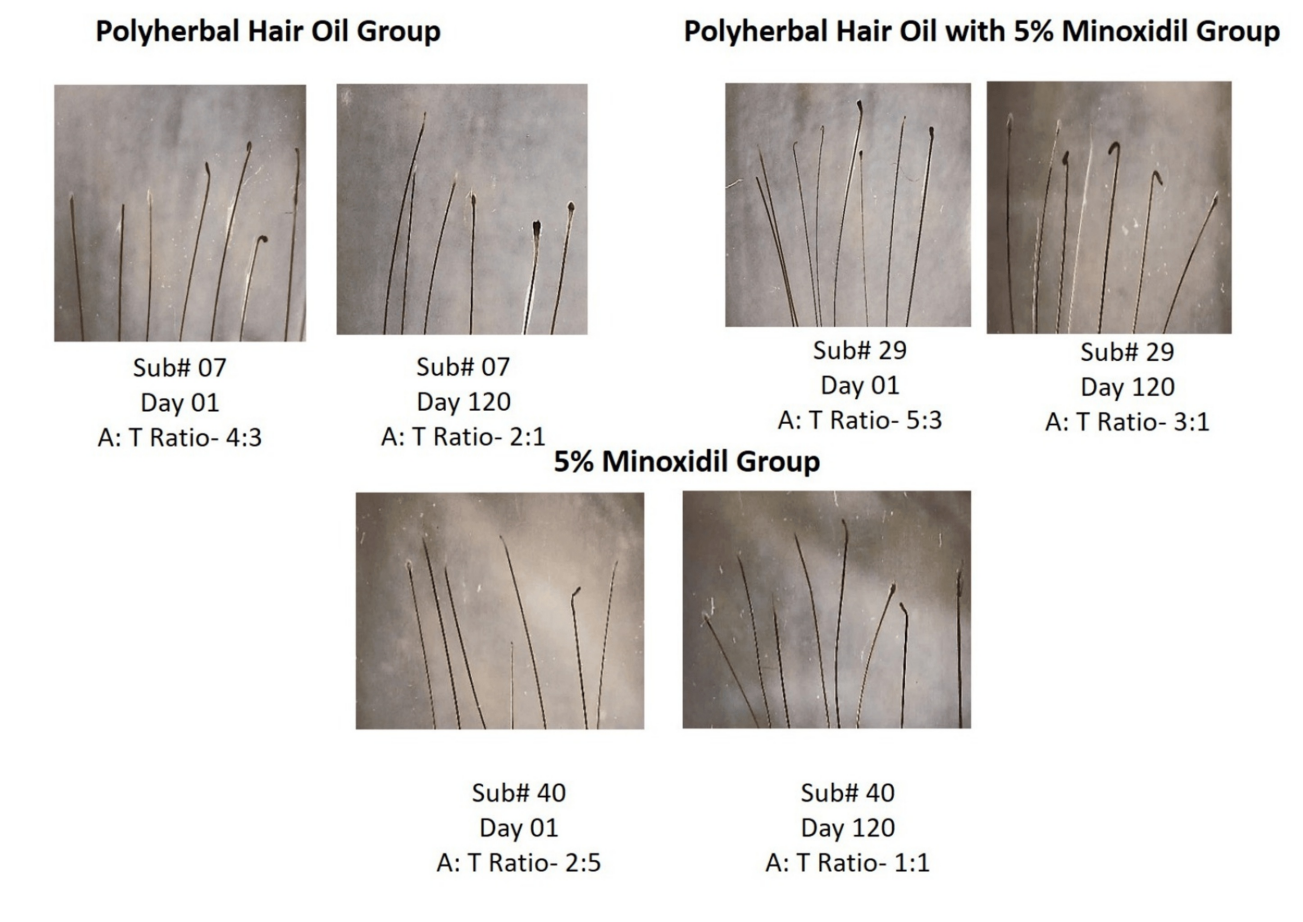
Anagen:Telogen Hairs Changes in the anagen to telogen ratio across study groups over the treatment period. Sub# denotes anonymized participant identification number. Sub# 07 represents a participant from the polyherbal hair oil group, Sub# 29 represents a participant from the polyherbal hair oil with minoxidil group, and Sub# 40 represents a participant from the minoxidil group. For subject# 31, Day 01 (4:3), Day 90 (2:1). For subject# 29, Day 01 (5:3), Day 90 (3:1). For subject# 31, Day 01 (2:5), Day 90 (1:1).

In the polyherbal hair oil group, the percentage of hairs in the telogen phase was found to be 61.31 ± 11.18 at baseline, which significantly decreased to 36.44 ± 11.21 on Day 120, showing a 38.27% reduction from baseline, with a statistically significant p-value of <0.0001. In the polyherbal with minoxidil group, the mean value was found to be 55.90 ± 13.74 at baseline; it decreased to 36.81 ± 12.93, a 29.38% change from baseline on Day 120, with a statistically significant p-value of <0.001. In the minoxidil group, the mean value was found to be 62.09 ± 14.39 at baseline, and it decreased to 35.45 ± 11.32, 41.49% on Day 120, with a statistically significant p-value of <0.0001 (Figure 6).

The 60-second hair comb test

Hair Fall Without Bulb

In the polyherbal hair oil group, the mean hair fall count was found to be 21.68 ± 10.73 at baseline, and it decreased to 8.68 ± 4.38 or 57.06% by Day 120, with a statistically significant p-value of <0.0001. In the polyherbal with minoxidil group, the mean hair fall count was 21.92 ± 7.19 at baseline, and it decreased to 9.16 ± 4.79 or 54.90% by Day 120, with a statistically significant p-value of <0.0001. In the minoxidil group, the mean hair fall count was 22.28 ± 7.33 at baseline, and it decreased to 9.32 ± 5.68 or 59.03% by Day 120, with a statistically significant p-value of <0.0001 (Table 4).

Hair Fall With Bulb

In the polyherbal hair oil group, the mean value was found to be 25.88 ± 9.64 at baseline, and it decreased to 12.80 ± 8.69 or 52.91% on Day 120 with a statistically significant p-value of <0.0001. In the polyherbal with minoxidil group, the mean value was found to be 25.76 ± 8.61 at baseline, and it decreased to 12.84 ± 6.52 or 51.39% on Day 120 with a statistically significant p-value of <0.0001. In the minoxidil group, the mean value was found to be 25.20 ± 7.89 at baseline, and it decreased to 12.84 ± 6.07 or 48.83% on Day 120 with a statistically significant p-value of <0.0001 (Table 4).

Total Number of Hair Fall

In the polyherbal hair oil group, the mean value was 47.56 ± 17.65 at baseline, and it decreased to 21.48 ± 11.11 or 55.20% by Day 120 with a statistically significant p-value of <0.0001. In the polyherbal with minoxidil group, the mean value was 47.68 ± 13.63 at baseline, and it decreased to 22.00 ± 9.92 or 53.65%; both time points also showed a statistically significant p-value of <0.0001. In the minoxidil group, the mean value was 47.48 ± 13.39 at baseline, and it decreased to 22.16 ± 10.69 or 53.71% by Day 120 with a statistically significant p-value of <0.0001 (Table 4).

Hair Shine

In the polyherbal hair oil group, the mean value was 1.62 ± 0.39 at baseline, and it increased to 2.17 ± 0.31 or 38.36% by Day 120 with statistically significant p-values of <0.0001. In the polyherbal with minoxidil group, the mean value was 1.58 ± 0.29 at baseline, and it increased to 2.03 ± 0.34 or 29.61% by Day 120 with statistically significant p-values of <0.0001. In the minoxidil group, the mean value was 1.66 ± 0.32 at baseline, and it increased to 2.01 ± 0.20 or 24.86% by Day 120 with a statistically significant p-value of <0.0001 (Table 4).

**Table 4:** Change in Evaluation Parameters from Baseline to the End of Study Duration Values are expressed as mean ± SD. %CFB = percentage change from baseline. Reported p-values correspond to within-group comparisons versus baseline. The statistical test (paired t-test) has been used. Participants who completed the final visit were included in the analysis, while data from intermediate visits were analyzed based on availability. SD: standard deviation

Variables	Visit day	Poly herbal oil					Poly herbal oil + minoxidil					Minoxidil				
Statistics		N	Mean ± SD	%CFB	p-value	T-value	N	Mean ± SD	%CFB	p-value	T-value	N	Mean ± SD	%CFB	p-value	T-value
Hair Fall without Bulb	Day 01	25	21.68 ± 10.73	-			25	21.92 ± 7.19	-			25	22.28 ± 7.33			
	Day 45	25	17.56 ± 10.74	22.15%	<0.0001	-4.25	22	18.05 ± 7.09	17.83%	<0.0001	-6.46	24	18.88 ± 6.97	16.15%	<0.0001	-7.57
	Day 90	24	13.88 ± 7.92	37.52%	<0.0001	-4.18	24	14.54 ± 5.84	34.36%	<0.0001	-8.51	25	14.40 ± 6.88	35.55%	<0.0001	-8.12
	Day 120	25	8.68 ± 4.38	57.06%	<0.0001	-4.35	25	9.16 ± 4.79	54.90%	<0.0001	-8.97	25	9.32 ± 5.68	59.03%	<0.0001	-11.18
Hair Fall with Bulb	Day 01	25	25.88 ± 9.64	-			25	25.76 ± 8.61	-			25	25.20 ± 7.89	-		
	Day 45	25	21.20 ± 8.69	18.69%	<0.0001	-8.97	22	22.14 ± 9.63	15.85%	<0.0001	-6.32	24	21.54 ± 5.77	14.18%	<0.0001	-6.32
	Day 90	24	17.71 ± 8.13	33.91%	<0.0001	-11.91	24	19.33 ± 8.25	26.75%	<0.0001	-8.55	25	17.88 ± 6.08	27.36%	<0.0001	-8.55
	Day 120	25	12.80 ± 8.69	52.91%	<0.0001	-10.55	25	12.84 ± 6.52	51.39%	<0.0001	-11.16	25	12.84 ± 6.07	48.83%	<0.0001	-11.16
Total Hair Fall	Day 01	25	47.56 ± 17.65	-			25	47.68 ± 13.63	-			25	47.48 ± 13.39	-		
	Day 45	25	38.76 ± 16.52	19.90%	<0.0001	-4.39	22	40.18 ± 13.48	16.40%	0.0001	-4.02	24	40.42 ± 11.75	15.60%	<0.0001	-4.30
	Day 90	24	31.58 ± 13.57	35.33%	<0.0001	-4.20	24	33.46 ± 11.59	31.26%	<0.0001	-4.29	25	32.28 ± 11.66	31.41%	<0.0001	-4.37
	Day 120	25	21.48 ± 11.11	55.20%	<0.0001	-4.37	25	22.00 ± 9.92	53.65%	<0.0001	-4.35	25	22.16 ± 10.69	53.71%	<0.0001	-4.38
Hair Shine	Day 01	25	1.62 ± 0.39	-			25	1.58 ± 0.29	-			25	1.66 ± 0.32	-		
	Day 45	25	1.83 ± 0.32	15.28%	<0.0001	-4.37	22	1.78 ± 0.30	10.91%	<0.0001	5.64	24	1.80 ± 0.27	9.68%	<0.0001	5.71
	Day 90	24	1.97 ± 0.32	26.28%	<0.0001	-4.29	24	1.82 ± 0.34	16.67%	<0.0001	7.78	25	1.90 ± 0.23	16.76%	<0.0001	-4.37
	Day 120	25	2.17 ± 0.31	38.36%	<0.0001	-4.37	25	2.03 ± 0.34	29.61%	<0.0001	10.54	25	2.01 ± 0.20	24.86%	<0.0001	-4.37
Hair Growth Rate	Day 1	25	249.88 ± 41.33	-	-	-	25	231.92 ± 50.44	-	-	-	25	233.84 ± 49.72	-	-	-
	Day 90	24	290.29 ± 39.57	17.05	<0.0001	7.20	24	286.21 ± 54.58	24.36	<0.0001	6.10	25	263.96 ± 48.59	14.13	<0.0001	5.18
% Anagen Hairs	Day 1	25	38.68 ± 11.19	-	-	-	25	44.10 ± 13.74	-	-	-	25	37.91 ± 14.39	-	-	-
	Day 120	25	63.56 ± 11.21	75.42	<0.0001	-4.37	25	63.19 ± 12.93	61.01	0.0002	4.81	25	64.55 ± 11.32	96.30	<0.0001	-4.29
% Telogen Hairs	Day 1	25	61.31 ± 11.18	-	-	-	25	55.90 ± 13.74	-	-	-	25	62.09 ± 14.39	-	-	-
	Day 120	25	36.44 ± 11.21	-39.64	<0.0001	-4.37	25	36.81 ± 12.93	-29.38	0.0002	-3.60	25	35.45 ± 11.32	-41.49	<0.0001	-4.29

The between-group statistical analysis demonstrated the comparative effects of the interventions on hair and scalp parameters throughout the study period (Table 5).

**Table 5:** Between-Group Statistical Comparison of Hair and Scalp Parameters Between-group: Independent t-test. Normality: Shapiro-Wilk test. Homogeneity: Levene’s test used.

Variables	Comparison	Day 45		Day 90		Day 120	
		p-value	t-value	p-value	t-value	p-value	t-value
Hair density	Poly herbal oil vs. poly herbal oil + minoxidil	0.923	-0.10	0.514	-0.10	0.372	-0.89
	Poly herbal oil vs. minoxidil	0.5502	-0.60	0.3662	-0.90	0.2637	-1.12
	Poly herbal oil + minoxidil vs. minoxidil	0.4354	-0.78	0.1370	-1.49	0.0369	-2.09
Hair thickness	Poly herbal oil vs. poly herbal oil + minoxidil	0.8762	-0.16	0.9820	-0.02	0.1067	-1.61
	Poly herbal oil vs. minoxidil	0.0003	-3.59	0.0003	-3.60	0.0080	-2.65
	Poly herbal oil + minoxidil vs. minoxidil	0.0048	-2.82	0.0013	-3.22	0.0004	-3.53
Skin Hydration	Poly herbal oil vs. poly herbal oil + minoxidil	0.4402	-0.78	0.8743	-0.16	0.1303	-1.54
	Poly herbal oil vs. minoxidil	0.5183	0.65	0.1093	1.63	0.0841	1.76
	Poly herbal oil + minoxidil vs. minoxidil	0.1920	8.24	0.1385	8.86	0.0063	12.65
ASFS scoring	Poly herbal oil vs. Poly herbal oil + minoxidil	0.5673	-0.58	0.8902	-0.14	0.3100	-1.02
	Poly herbal oil vs. minoxidil	0.0308	-2.16	0.0181	-2.46	0.1405	-1.50
	Poly herbal oil+ minoxidil vs. minoxidil	0.0928	-1.68	0.0266	-2.30	0.0085	-2.74
GSS	Poly herbal oil vs. poly herbal oil + minoxidil	0.0099	-2.65	0.0597	-1.59	0.3100	-2.16
	Poly herbal oil vs. minoxidil	0.0308	-3.34	0.0080	-2.60	<0.0001	-5.12
	Poly herbal oil + minoxidil vs. minoxidil	0.0928	-0.53	0.1837	-1.85	<0.0001	-3.91
Hair fall without bulb	Poly herbal oil vs. poly herbal oil + minoxidil	0.7105	-0.37	0.6945	-0.40	0.3308	-0.97
	Poly herbal oil vs. minoxidil	0.3953	-0.86	0.8407	-0.20	0.5663	-0.57
	Poly herbal oil + minoxidil vs. minoxidil	0.6708	-0.43	0.8481	0.19	0.6617	-0.44
Hair fall with bulb	Poly herbal oil vs. poly herbal oil + minoxidil	0.1645	-1.41	0.0657	-1.89	0.9252	-0.09
	Poly herbal oil vs. minoxidil	0.2501	-0.96	0.3410	-0.43	0.6659	-1.15
	Poly herbal oil + minoxidil vs. minoxidil	0.7810	0.50	0.6177	-0.35	0.7273	-0.28
Total no. of hairs	Poly herbal oil vs. poly herbal oil + minoxidil	0.1601	-1.43	0.2552	-1.15	0.8985	-0.13
	Poly herbal oil vs. minoxidil	0.1878	-1.34	0.5123	-0.66	0.7967	-0.26
	Poly herbal oil + minoxidil vs. minoxidil	0.9675	0.04	0.8110	0.24	0.9054	-0.12
Hair shine	Poly herbal oil vs. Poly herbal oil+ minoxidil	0.2160	-1.24	0.0421	2.09	0.1643	1.41
	Poly herbal oil vs. minoxidil	0.0747	-1.78	0.0107	-2.55	0.0070	-2.70
	Poly herbal oil + minoxidil vs. minoxidil	0.5233	-0.64	0.5753	-0.56	0.0894	-1.70

General Appearance of Hair

Hair Volume

By Day 120, the polyherbal hair oil group showed 68% full hair volume and 32% medium volume, with no cases of small volume. The combination group (polyherbal with minoxidil) demonstrated 64% full volume, 32% medium volume, and 4% small volume. In comparison, the minoxidil group showed 44% full volume and 56% medium volume, with no small volume reported.

Hair Plasticity

By Day 120, no meaningful changes in hair plasticity were observed across groups. The polyherbal group showed 88% flat and 12% waved hair, the combination group demonstrated 84% flat and 16% waved hair, and the minoxidil group exhibited no observable change during the study period.

Hair Density

By Day 120, the polyherbal group showed 80% dense hair with only 20% exhibiting thinning or shedding. In the combination group, thinning reduced markedly to 28% from baseline. The minoxidil group demonstrated 72% dense hair with 28% still showing thinning, indicating substantial but comparatively delayed improvement.

Hair Shininess

By Day 120, 100% of participants in the polyherbal group achieved good hair shininess. The combination group showed near-complete improvement, with only 8% remaining average. In the minoxidil group, 76% exhibited good shininess, while 24% remained average, indicating comparatively gradual improvement.

Hair Smoothness

By Day 120, 92% of participants in the polyherbal group achieved good hair smoothness, compared to 88% in the combination group. In the minoxidil group, 68% demonstrated good smoothness, while 32% remained average, indicating comparatively gradual improvement.

Hair Reflection

By Day 120, 100% of participants in both the polyherbal and combination groups exhibited shiny hair reflection. In the minoxidil group, 92% demonstrated shiny reflection, while 8% retained blunt reflection, indicating comparatively slower improvement. 

General appearance of scalp

Scalp Itchiness

In the polyherbal hair oil group and polyherbal with minoxidil group, no itchiness was reported in 100% of the participants by Day 120. In the minoxidil group, no itchiness was reported in 96% of participants by Day 120.

Scalp Redness

Across all products, no redness was observed by Day 120.

Scalp Roughness

In the polyherbal hair oil group, by Day 120, 96% participants reported no roughness. In the polyherbal with minoxidil group, 100% participants reported no roughness. In the minoxidil group, 80% had no roughness.

Scaliness

In the polyherbal hair oil group, 100% participants reported no scaliness. In the polyherbal with minoxidil group, 96% participants reported no scaliness. In the minoxidil group, 88% participants reported no scaliness.

Dryness

In the polyherbal hair oil group, no dryness was reported in 80% of participants by Day 120. In the polyherbal with minoxidil group, 88% participants reported no dryness. In the minoxidil group, 56% had no roughness.

Participant perception questionnaire

On Day 120, the test products were well tolerated, with all participants reporting no irritation-related reactions such as redness, dryness, itching, or burning of the scalp. Participative assessments indicated favorable cosmetic and efficacy outcomes, with the majority of participants reporting improvements in hair softness, silkiness, and shine, primarily to a moderate or large extent. Perceived efficacy in reducing gray hair was also reported, with most participants noting moderate improvement and a smaller proportion reporting a large extent of benefit. Improvements in hair thickness and density, as well as a reduction in hair fall, were predominantly reported to a moderate or large extent. Additionally, participants observed enhancements in overall scalp health and reduction in dandruff, largely to moderate or large extents. Overall satisfaction with the test products was high, with the majority of participants expressing satisfaction to a large extent at the end of the study period (Appendices).

Scanning electron microscopy

The polyherbal hair oil group demonstrated a noticeable improvement in hair cuticle condition following the product usage period. The baseline images revealed a mildly lifted cuticle layer, roughness, and initial signs of weathering that are normal responses to normal environmental and mechanical stress. The micrographs after product use revealed a smoother cuticle layer structure with improved edge alignment of the scales. The reduction in surface irregularities and improved cohesion of the cuticle layer indicate improved integrity of the structure of the hair shaft. The micrographs revealed improved smoothness of the cuticle layer, which indicates a positive effect of the product on hair health.

In the case of the polyherbal hair oil with minoxidil group, consistent improvement in cuticle morphology was observed in both subjects after the use of the product. The hair shafts were observed to have mild surface roughness and lifting of the edges of the cuticle scales, and evidence of initial signs of weathering due to routine physical and environmental stress. However, the hair shafts showed smoothness and uniformity in the cuticle scale arrangement after the use of the product. There was also less lifting of the edges of the cuticle scales and fewer irregularities on the hair shaft surface.

**Figure 7:**
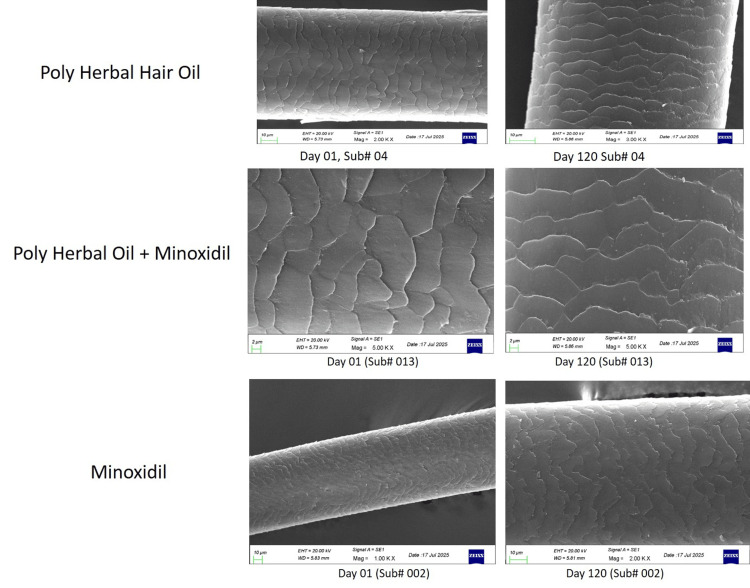
Scanning Electron Microscopy Findings Left panel: baseline (Day 01) image showing hair cuticle morphology before test product usage. Right panel: post-treatment (Day 120) image showing changes in hair cuticle structure following product use. Sub# 14 represents a participant from the polyherbal hair oil group, Sub# 13 represents a participant from the polyherbal hair oil with minoxidil group, and Sub# 02 represents a participant from the minoxidil group.

For the minoxidil group, positive changes were observed in cuticle morphology after the product usage period. The hair shafts were observed to be slightly rough on the surface and had misoriented cuticle scales at baseline, indicating initial signs of weathering and mechanical stress. After the product usage period, the hair shaft surface appeared smoother with well-aligned cuticle scales and less lifting around the edges. The cuticle scales were observed to be uniform and cohesive, suggesting stabilization of these outer cuticle layers. Both individuals showed positive changes in cuticle integrity and surface texture after the product usage period (Figure 7).

A subgroup analysis was conducted as an equal number of participants were enrolled in each group. The analysis was performed based on two conditions: androgenetic alopecia and hair fall, both having gray hairs. This helped to evaluate the product response separately in participants with androgenetic alopecia and those primarily experiencing hair fall during the study period (Tables 6-7).

**Table 6:** Subgroup Analysis of Evaluation Parameters from Baseline to the End of Study Duration Subgroup analysis of evaluation parameters, such as hair density, hair thickness, scalp hydration, ASFS scoring, and GSS from baseline to the end of study duration. The statistical test (paired t-test) was used. Participants who completed the final visit were included in the analysis, while data from intermediate visits were analyzed based on availability. ASFS: Adherent Scalp Flaking Score; GSS: Graying Severity Score; SD: standard deviation

Variables	Visit day	Poly herbal oil					Poly herbal oil + minoxidil					Minoxidil				
		N	Mean ± SD	%CFB	p- value	t-value	N	Mean ± SD	%CFB	p- value	t-value	N	Mean ± SD	%CFB	p- value	t-value
Hair Density (hairs/cm²)	AGA group															
	Day 01	12	225.33 ± 29.74	-			13	240.54 ± 45.38	-			13	225.31 ± 24.22	-		
	Day 45	12	237.00 ± 27.84	5.38%	<0.0001	9.11	11	263.82 ± 32.43	4.65%	<0.0001	9.52	13	235.62 ± 23.26	4.67%	<0.0001	13.98
	Day 90	11	249.45 ± 26.44	11.63%	<0.0001	12.92	12	269.17 ± 34.99	14.90%	<0.0001	7.02	13	246.38 ± 24.39	9.51%	<0.0001	8.55
	Day 120	12	262.00 ± 20.71	17.02%	<0.0001	12.41	13	284.23 ± 33.98	20.05%	<0.0001	9.23	13	257.38 ± 24.15	14.49%	<0.0001	10.24
	Hair fall group															
	Day 01	13	253.69 ± 32.03	-			12	226.33 ± 42.16	-			12	226.92 ± 35.32	-		
	Day 45	13	263.08 ± 32.09	3.77%	<0.0001	8.66	11	236.36 ± 44.21	4.21%	<0.0001	75.28	11	233.91 ± 35.84	4.31%	<0.0001	60.04
	Day 90	13	273.31 ± 27.99	8.08%	<0.0001	8.66	12	248.33 ± 40.83	10.32%	<0.0001	10.25	12	250.50 ± 34.32	10.96%	0.0018	4.09
	Day 120	13	292.00 ± 37.53	15.52%	0.0002	5.26	12	260.67 ± 39.02	16.22%	<0.0001	11.96	12	258.42 ± 35.27	14.55%	0.0004	5.08
Hair Thickness (µm)	AGA group															
	Day 1	12	13.50 ± 2.07	-			13	9.62 ± 2.60	-			13	11.17 ± 1.54	-		
	Day 45	12	14.58± 1.93	8.41%	<0.0001	13	11	11.09 ± 2.26	16.48%	<0.0001	8.96	13	12.38 ± 1.71	5.23%	0.0009	4.38
	Day 90	11	15.82 ± 1.94	19.19%	<0.0001	9.93	12	12.17 ± 2.48	30.74%	<0.0001	8.98	13	13.38 ± 1.89	13.66%	<0.0001	8.95
	Day 120	12	17.08 ± 1.88	27.67%	<0.0001	12.46	13	13.69 ± 2.21	48.16%	<0.0001	10.2	13	14.31 ± 2.02	21.70%	<0.0001	8.72
	Hair fall group															
	Day 01	13	12.92 ± 2.02	-			12	12.83 ± 1.70	-			12	12.33 ± 2.19	-		
	Day 45	13	14.08± 2.02	9.20%	<0.0001	11.08	11	13.82 ± 1.72	6.37%	<0.0001	6.71	11	13.00 ± 2.14	4.87%	0.0251	2.63
	Day 90	13	15.08 ± 2.10	17.08%	<0.0001	14	12	14.92 ± 1.93	16.45%	<0.0001	8.02	12	13.67 ± 2.06	11.58%	0.0001	5.93
	Day 120	13	16.08 ± 2.02	25.15%	<0.0001	16.51	12	16.50 ± 1.83	29.05%	<0.0001	14.31	12	14.83 ± 2.29	21.58%	0.0001	6.27
Scalp hydration (µm/day)	AGA group															
	Day 01	12	9.79 ± 1.17	-			13	9.83 ± 1.59	-			13	9.57 ± 0.89	-		
	Day 45	12	10.81 ± 1.04	10.77%	<0.0001	6.87	11	11.02 ± 1.62	12.10%	0.0002	5.71	13	10.50 ± 0.93	9.91%	<0.0001	6.76
	Day 90	11	11.34 ± 1.47	17.06%,	<0.0001	6.76	12	11.75 ± 1.44	20.54%	0.0001	5.65	13	11.07 ± 1.00	15.86%	<0.0001	9.28
	Day 120	12	12.06 ± 1.20	23.73%,	<0.0001	9.52	13	12.43 ± 1.64	27.61%	<0.0001	7.94	13	11.63 ± 1.08	21.80%	<0.0001	10.18
	Hair fall group															
	Day 01	13	9.56 ± 0.94	-			12	9.58 ± 1.49	-			12	9.85 ± 1.34	-		
	Day 45	13	10.58 ± 1.28	10.59%	<0.0001	6.2	11	10.61 ± 1.28	13.43%	0.0002	5.66	11	10.63 ± 1.05	10.02%	0.0014	4.39
	Day 90	13	11.45 ± 1.02	20.14%,	<0.0001	9.9	12	11.33 ± 1.35	19.55%	<0.0001	6.92	12	11.26 ± 1.19	15.08%	<0.0001	7.22
	Day 120	13	12.11 ± 0.97	27.01%,	<0.0001	15.64	12	12.64 ± 1.66	33.22%	<0.0001	10.24	12	11.89 ± 1.07	21.76%	<0.0001	9.16
ASFS scoring	AGA group															
	Day 01	12	22.33 ± 7.18	-			13	20.31 ± 6.73	-			13	20.15 ± 7.23	-		
	Day 45	12	17.00 ± 3.86	20.19%	0.0046	-3.55	11	15.09 ± 3.94	19.61%	0.0119	-3.07	13	17.54 ± 4.70	9.08%	0.0311	-2.44
	Day 90	11	16.00 ± 3.79	25.44%	0.0028	-3.93	12	13.83 ± 7.74	37.03%	0.0009	-4.52	13	14.92 ± 3.43	19.71%	0.0054	-3.39
	Day 120	12	9.33 ± 5.21	56.79%	0.0001	-6	13	3.85 ± 3.21	81.81%	<0.0001	-9.37	13	9.38 ± 6.02	50.99%	0.0002	-5.3
	Hair fall group															
	Day 01	13	22.92 ± 4.87	-			12	25.17 ± 6.29	-			12	22.17 ± 5.01	-		
	Day 45	13	16.31 ± 3.15	26.35%	0.0005	-4.72	11	18.36 ± 2.94	22.33%	0.0014	-4.36	11	17.64 ± 2.80	15.20%	0.0032	-3.85
	Day 90	13	11.85 ± 4.51	46.18%	<0.0001	-6.39	12	14.83 ± 5.22	38.57%	0.0003	-5.3	12	17.00 ± 4.39	22.90%	<0.0001	-6.49
	Day 120	13	6.92 ± 4.59	70.02%	<0.0001	-11.03	12	7.83 ± 6.00	69.46%	<0.0001	-8.78	12	9.33 ± 5.74	58.28%	<0.0001	-7.2
GSS	AGA group															
	Day 01	12	12.00 ± 3.16	-			13	12.15 ± 2.34	-			13	12.92 ± 2.43	-		
	Day 45	12	11.17 ± 3.01	6.34%	0.0341	-2.42	11	11.64 ± 2.69	2.03%	0.3409	-1	13	12.85 ± 2.38	0.51%	0.3370	-1
	Day 90	11	10.18 ± 3.06	12.33%	0.0154	-2.92	12	11.00 ± 3.07	9.59%	0.0039	-3.63	13	12.54 ± 2.70	3.43%	0.0544	-2.13
	Day 120	12	9.33 ± 3.11	23.64%	<0.0001	-7.5	13	10.69 ± 3.28	13.62%	0.0019	-3.96	13	12.62 ± 2.69	2.77%	0.1039	-1.76
	Hair fall group															
	Day 01	13	11.85 ± 2.76	-			12	12.25 ± 2.18	-			12	11.92 ± 3.09	-		
	Day 45	13	11.00 ± 3.00	7.59%	0.0205	-2.67	11	12.00 ± 2.24	0.76%	0.3409	-1	11	11.55 ± 2.98	0.61%	0.3409	-1
	Day 90	13	10.15 ± 2.91	14.65%	0.0011	-4.25	12	11.33 ± 2.39	7.81%	0.0006	-4.75	12	11.08± 3.06	6.64%	0.0538	-2.16
	Day 120	13	9.15 ± 3.56	24.68%	0.0001	-6.06	12	10.08 ± 2.71	18.56%	0.0001	-6.29	12	11.33 ± 3.45	5.93%	0.0463	-2.24

**Table 7:** Subgroup Analysis of Evaluation Parameters from Baseline to the End of Study Duration Subgroup analysis of evaluation parameters such as hair fall without bulb, hair fall with bulb, total hair fall, hair shine, A:T ratio (anagen ratio), A:T ratio (telogen ratio) from baseline to the end of study duration, %CFB = percentage change from baseline. The statistical test (paired t-test) was used. Participants who completed the final visit were included in the analysis, while data from intermediate visits were analyzed based on availability.

Variables	Visit day	Poly herbal oil					Poly herbal oil + minoxidil					Minoxidil				
		N	Mean ± SD	%CFB	P-value	t-value	N	Mean ± SD	%CFB	p-value	t-value	N	Mean ± SD	%CFB	p-value	t-value
Hair fall without bulb	AGA group															
	Day -4	12	18.42 ± 8.18	-			13	22.23 ± 8.09	-			13	22.00 ± 8.54	-		
	Day 45	12	13.75 ± 8.06	28.09%	0.0002	-5.51	11	18.09 ± 7.99	19.46%	0.0012	-4.48	13	18.54 ± 7.78	16.48%	<0.0001	-6.9
	Day 90	11	11.00 ± 7.25	44.00%	<0.0001	-6.42	12	14.83 ± 5.54	33.02%	0.0006	-4.71	13	13.77 ± 5.80	36.06%	<0.0001	-6.89
	Day 120	12	6.67 ± 3.65	62.66%	<0.0001	-6.19	13	9.85 ± 5.21	48.10%	0.0004	-4.88	13	8.31 ± 4.97	62.99%	<0.0001	-8.24
	Hair fall group															
	Day -4	13	24.69 ± 12.18	-			12	21.58 ± 6.42	-			12	22.58 ± 6.11	-		
	Day 45	13	21.08 ± 11.98	16.67%	0.0003	-5.01	11	18.00 ± 6.47	16.21%	0.0007	-4.85	11	19.27 ± 6.23	15.77%	0.0019	-4.19
	Day 90	13	16.31 ± 7.90	32.05%	0.0003	-5.08	12	14.25 ± 6.36	35.71%	<0.0001	-10.17	12	15.08 ± 8.10	35.00%	0.0007	-4.68
	Day 120	13	10.54 ± 4.29	51.89%	0.0006	-4.61	12	8.42 ± 4.40	62.26%	<0.0001	-11	12	10.42 ± 6.39	54.73%	<0.0001	-7.35
Hair fall with bulb	AGA group															
	Day -4	12	23.67 ± 10.26	-			13	27.15 ± 6.80	-			13	25.92 ± 9.43	-		
	Day 45	12	19.25 ± 9.12	19.00%	0.0002	-5.64	11	23.55 ± 7.37	13.93%	0.0002	-5.83	13	22.00 ± 6.28	12.78%	0.0011	-3
	Day 90	11	16.27 ± 9.27	34.89%	<0.0001	-6.75	12	22.08 ± 7.69	20.89%	0.0005	-4.88	13	19.23 ± 6.34	24.36%	0.0004	-4.87
	Day 120	12	9.67 ± 5.82	59.46%	<0.0001	-7.74	13	14.77 ± 6.06	45.91%	<0.0001	-9.07	13	14.08 ± 7.40	46.62%	<0.0001	-8.55
	Hair fall group															
	Day -4	13	27.92 ± 8.94	-			12	24.25 ± 10.32	-			12	24.42 ± 6.13	-		
	Day 45	13	23.00 ± 8.22	18.40%	<0.0001	-6.84	11	20.73 ± 11.67	17.77%	0.0044	-3.66	11	21.00 ± 5.35	15.83%	0.0001	-6.47
	Day 90	13	18.92 ± 7.17	33.07%	<0.0001	-10.09	12	16.58 ± 8.16	32.61%	<0.0001	-7.65	12	16.42 ± 5.70	30.60%	0.0013	-4.27
	Day 120	13	15.69 ± 10.05	46.85%	<0.0001	-7.04	12	10.75 ± 6.59	57.32%	<0.0001	-6.9	12	11.50 ± 4.12	51.23%	<0.0001	-7.25
Total hair fall	AGA group															
	Day -4	12	42.08 ± 14.43	-			13	49.38 ± 12.14	-			13	47.92 ± 16.31	-		
	Day 45	12	33.00 ± 13.64	23.06%	<0.0001	-11.07	11	41.64 ± 10.50	16.01%	<0.0001	-8.61	13	40.54 ± 13.45	15.05%	0.0004	-4.88
	Day 90	11	27.27 ± 14.14	39.65%	<0.0001	-10.29	12	36.08 ± 10.77	28.44%	<0.0001	-7.72	13	33.00 ± 10.55	29.95%	<0.0001	-6.73
	Day 120	12	16.33 ± 7.48	61.54%	<0.0001	-8.85	13	24.62 ± 9.64	48.10%	<0.0001	-7.03	13	22.38 ± 11.72	54.33%	<0.0001	-9.71
	Hair fall group															
	Day -4	13	52.62 ± 19.35	-			12	45.83 ± 15.40	-			12	47.00 ± 10.02	-		
	Day 45	13	44.08 ± 17.65	16.98%	<0.0001	-11.69	11	38.73 ± 16.33	16.80%	0.0007	-4.81	11	40.27 ± 10.04	16.24%	<0.0001	-11.34
	Day 90	13	35.23 ± 12.44	31.68%	<0.0001	-7.27	12	30.83 ± 12.23	34.07%	<0.0001	-10.9	12	31.50 ± 13.19	33.00%	0.0006	-4.75
	Day 120	13	26.23 ± 12.03	49.36%	<0.0001	-8.09	12	19.17 ± 9.81	59.66%	<0.0001	-9.23	12	21.92 ± 9.97	53.03%	<0.0001	-7.98
Hair shine	AGA group															
	Day 01	12	1.63 ± 0.35	-	-	-	13	1.61 ± 0.26	-	-	-	13	1.73 ± 0.34	-		
	Day 45	12	1.82 ± 0.29	12.99%	0.0002	5.34	11	1.81 ± 0.20	9.15%	0.0085	3.26	13	1.84 ± 0.32	6.97%	0.0004	4.8
	Day 90	11	1.95 ± 0.32	23.28%	<0.0001	7.76	12	1.79 ± 0.26	13.67%	0.0008	4.61	13	1.92 ± 0.31	12.06%	<0.0001	7.48
	Day 120	12	2.14 ± 0.32	33.57%	<0.0001	7.39	13	2.00 ± 0.33	24.33%	<0.0001	7.03	13	2.07 ± 0.25	22.10%	<0.0001	6.32
	Hair fall group															
	Day 01	13	1.61 ± 0.44	-	-	-	12	1.55 ± 0.32	-			12	1.58 ± 0.28	-		
	Day 45	13	1.84 ± 0.36	17.40%	0.0008	4.47	11	1.75 ± 0.37	12.66%	0.0008	4.71	11	1.76 ± 0.21	12.88%	0.0031	3.87
	Day 90	13	1.99 ± 0.33	28.82%	<0.0001	5.79	12	1.86 ± 0.42	19.67%	<0.0001	6.68	12	1.86 ± 0.11	21.85%	0.0016	4.17
	Day 120	13	2.19 ± 0.31	42.78%	<0.0001	7.24	12	2.07 ± 0.36	35.34%	<0.0001	8.33	12	1.94 ± 0.10	27.84%	0.0014	4.25
Hair growth rate (µm/day)	AGA Group															
	Day 01	12	240.92 ± 37.87	-	-	-	13	230.15 49.60	-	-	-	13	229.46 51.62	-	-	-
	Day 90	11	283.82 ± 36.07	18.52	0.0004	5.13	12	274.92 51.31	19.37	0.0017	4.11	13	261.92 47.72	15.70	0.0024	3.82
	Hair fall group															
	Day 01	13	258.15 ± 44.13	-	-	-	12	233.83 ± 53.47	-	-	-	12	238.58 ± 49.40	-	-	-
	Day 90	13	295.77 ± 42.96	15.80	0.0004	4.88	12	297.50 ± 57.61	29.36	0.0007	4.68	12	266.17 ± 51.55	12.43	0.0064	3.36
% Anagen hairs	AGA group															
	Day 01	12	37.68 ± 11.73	-	-	-	13	45.06 ± 14.64	-	-	-	13	39.25 ± 13.00	-	-	-
	Day 120	12	64.88 ± 10.24	82.68%	<0.0001	8.15	13	65.64 ± 10.04	61.14%	0.0006	4.66	13	62.18 ± 8.43	74.95%	<0.0001	7.26
	Hair fall group															
	Day 1	13	39.61 ± 11.05	-			12	43.05 ± 13.26	-			12	36.45 ± 16.23	-		
	Day 120	13	62.34 ± 12.33	68.72%	<0.0001	6.10	12	60.54 ± 15.49	60.86%	0.0283	2.52	12	67.12 ± 13.71	119.44%	<0.0001	6.19
% Telogen hairs	AGA group															
	Day 01	12	62.32 ± 11.73	-			13	54.93 ± 14.64	-			13	60.75 ± 12.99	-		
	Day 120	12	35.12 ± 10.24	42.99%	<0.0001	-8.15	13	34.36 ± 10.04	34. 41%	0.0006	-4.66	13	37. 82 ± 8.43	36.36%	<0.0001	7.26
	Hair fall group															
	Day 01	13	60.39 ± 11.05	-			12	56.94 ± 13.26	-			12	63.54 ± 16.22	-		
	Day 120	13	37.65 ± 12.33	36.56%	<0.0001	-6.1	12	39.46 ± 15.50	23.93%	0.0284	-2.52	12	32.88 ± 13.71	47.05%	<0.0001	-6.19

## Discussion

This clinical study provides an evaluation of a polyherbal hair oil and its combination with minoxidil and minoxidil alone, assessing their benefits on hair growth rate, hair density, hair thickness, scalp health, and hair morphology over a period of 120 days. These findings reflected that all three products resulted in clinically and statistically significant improvements across multiple hair parameters.

Instrumental evaluations revealed significant and continuous improvements in hair thickness, hair density, and hair growth rate across all groups. Notably, the polyherbal hair oil, both as monotherapy and in combination with minoxidil, demonstrated greater increases in hair thickness and density over the duration. These findings suggest that the polyherbal formulation may show its multi-targeted benefits on hair growth and related parameters, supporting not only its role in hair thickness and density but also in the reduction of canities effect. The observed increase in the A:T ratio and hair strength across all groups further supports enhanced follicular activity, with the magnitude of improvement indicating a favorable shift toward the growth phase of the hair cycle by Day 120.

Hair fall reduction, assessed through the 60-second hair combing test and differentiation of hairs with and without bulbs, showed marked and statistically significant decreases in all product arms. The progressive reduction in hair shedding, particularly evident in the polyherbal-containing groups, suggests improved hair anchorage and follicular stability. Reduction in hair fall with bulbs may reflect normalization of the hair cycle and reduced premature transition to the telogen phase, while reductions in hair fall without bulbs likely indicate improved hair shaft strength and reduced breakage. The consistency of these findings across multiple hair fall endpoints strengthens the clinical relevance of the observed effects. Parameters associated with scalp, including scalp appearance and hydration flake assessment through scalp keratin assessment, illustrated significant improvements over the entire study duration. Polyherbal hair oil showed higher improvement in scalp keratin and hydration compared to minoxidil, indicating enhanced scalp hydration.

The outcomes are a matter of interest, as scalp health is an important criterion for the evaluation of hair fall, and maintenance of hair growth. The graying severity score improved over the study duration, with a greater effect observed in the polyherbal hair oil group compared with the other test products. The amplitude of reduction in canity severity was consistent, which demonstrated the effect of the polyherbal hair oil in improving hair pigmentation.

Clinical assessment of the general appearance of hair and scalp further supported the instrumental findings. Participants receiving the polyherbal hair oil and its combination exhibited improvements in hair density, shine, volume, smoothness, and overall hair appearance, along with improvement in scalp, roughness, scaliness, dryness, and itchiness. These improvements are helpful, as they directly affect the hair oil acceptability, user satisfaction, and long-term product compliance. The cosmetic benefits provided by polyherbal-based hair oil and combination therapy may help to address some of the patient compliance problems associated with conventional pharmacological therapies.

The scanning electron microscopy results provided supportive evidence for the product-related changes in the hair. The images acquired after product application revealed improved cuticle alignment, reduced lifting, and surface smoothness of the hair shaft for all groups. More consistent restoration of cuticles was observed for the polyherbal formulation. These changes are indicative of improved surface integrity and reduced damage to the hair shaft. These changes are in conformity with the improved smoothness, gloss, and combability of the hair.

From an integrative perspective, the beneficial effects of the polyherbal hair oil appear consistent with those of the Ayurvedic approach of hair care through scalp conditioning and nourishment. The polyherbal formulation of the hair oil may have a synergistic effect on hair growth through its multiple active ingredients targeting various pathways simultaneously, including antioxidant activity, anti-inflammatory effects, conditioning of the scalp, and strengthening of hair follicles. In contrast, minoxidil appears to exert its hair growth-promoting activity through vasodilatory and anagen phase-prolonging mechanisms.

The results of the study indicated a good safety profile of formulations in all groups. There were no reports of serious adverse events related to the product. The lack of irritancy responses in the scalp in the polyherbal hair oil formulation highlights its safety profile. Hair loss conditions are long-term problems, and the use of a formulation over a long period of time assumes significance. Ayurvedic formulations have traditionally followed a holistic approach in medicine. These formulations have now come under the scanner of modern science with a view to proving their efficacy through scientific means. The use of advanced technology in formulation development helps in the assessment of Ayurveda [22].

Past in vitro studies have already revealed the protective and strengthening benefits of Neeli-Bhringraj-based hair oil on hair strands. In the in vitro study on the evaluation of hair breakage and tensile strength of the Neeli Bhringraj oil on the hair strands, Neeli Bhringraj oil was found to possess better protective benefits against hair breakage compared to the vehicle control. There was a significant reduction in the percentage of short-length fiber breakage and a shift toward long-length hair strands. This proves the enhanced tensile strength of the hair strands after the application of the Neeli Bhringraj oil. The enhanced strength and protective benefits against mechanical stress on the hair strands are a scientific validation of the traditional benefits of Neeli Bhringraj oil on hair strands. This study provides a scientific validation for the evaluation of Neeli Bhringraj oil as a non-synthetic intervention in the prevention of hair fall [23].

The results of the present study are in accordance with those of previously published experimental and clinical data related to Neeli-Bhringraj and its individual major constituents. A controlled study showed that *Eclipta alba*, commonly known as Bhringraja, a major component of Neeli-Bhringraj, was found to promote hair growth by enhancing the ratio of anagen hair follicles and follicular size [24]. In a clinical study on the efficacy of polyherbal hair oil formulations containing Neeli and Bhringraj extracts, Choudhary found that hair fall was significantly reduced along with an improvement in hair texture and scalp health after topical application. These results are similar to those obtained in this study on Neeli-Bhringraj-based polyherbal hair growth promoters that showed consistent improvement in hair growth parameters, reduction in hair fall, and improvement in scalp health and hair texture. A previous study showed that topical minoxidil demonstrated hair regrowth in patients with androgenetic alopecia after 12 months, with response correlating to systemic absorption levels, while no significant regrowth was observed in patients with alopecia areata [25]. A study showed that oral and topical minoxidil demonstrated comparable efficacy in improving hair density in androgenetic alopecia. Oral minoxidil (5 mg once daily) did not demonstrate significant superiority over topical minoxidil (5% twice daily) in improving hair density in men with androgenetic alopecia over 24 weeks. However, oral minoxidil showed a modest advantage in vertex improvement, with a higher incidence of systemic adverse effects such as hypertrichosis [26].

The study has some limitations, and it is important that we take them into consideration. The study population is small, and it is a single-center study, which may affect the results. Second, though many objective and subjective parameters were studied, a long-term study would be beneficial in confirming the sustained benefits of the polyherbal formulation. In conclusion, the results of the study confirm the clinical benefits of polyherbal hair oil formulation as a potent and safe solution for the management of hair fall, hair thinning, and scalp disorders. The polyherbal hair oil with minoxidil was found to be effective in all parameters, and it is a potential solution for integrative hair care. 

## Conclusions

*Neeli Bhringadi *hair oil was well tolerated and demonstrated favorable effects on multiple hair and scalp parameters over the 120-day study period. Improvements were observed in measures related to hair growth, hair quality, clinical graying severity, and scalp condition, indicating its potential role in supporting overall hair and scalp health. The formulation may contribute to the maintenance of healthy hair and scalp characteristics and could serve as a valuable component of an integrative hair care regimen. These findings provide preliminary clinical evidence supporting the use of this Ayurvedic polyherbal formulation in individuals experiencing hair fall, androgenetic alopecia, and premature hair graying.

## Appendices

Sr. no

Questionnaire

Response

1.        Did you experience any irritation reactions, such as redness, dryness, itchiness, and burning sensation, of the scalp upon usage of the test products?

1 = Not at all

2 = To a small extent

3 = To some extent 

4 = To a moderate extent

5 = To a large extent

2.        Do you feel that your hair becomes soft, silky, and shiny after usage of the test products?

1 = Not at all

2 = To a small extent

3 = To some extent 

4 = To a moderate extent

5 = To a large extent

3.        Do you feel the test products are effective in the reduction of gray hair?

1 = Not at all

2 = To a small extent

3 = To some extent 

4 = To a moderate extent

5 = To a large extent

4.        Do you feel that the test products are effective in improving hair thickness and density?

1 = Not at all

2 = To a small extent

3 = To some extent 

4 = To a moderate extent

5 = To a large extent

5.        Do you feel that the test products are effective in reducing hair fall?

1 = Not at all

2 = To a small extent

3 = To some extent 

4 = To a moderate extent

5 = To a large extent

6.        Do you feel that the test products effectively provide the healthier-looking hair/scalp?

1 = Not at all

2 = To a small extent

3 = To some extent 

4 = To a moderate extent

5 = To a large extent

7.        Do you feel that the test products reduce dandruff?

1 = Not at all

2 = To a small extent

3 = To some extent 

4 = To a moderate extent

5 = To a large extent

8.        How satisfied are you with the usage of test products?

1 = Not at all

2 = To a small extent

3 = To some extent 

4 = To a moderate extent

5 = To a large extent
